# Event and Comment

**Published:** 1908-12-15

**Authors:** 


### DENTAL REGISTER.

Vol. LXII. December 15, 1908. No. 12.

### EVENT AND COMMENT.

### Healthful Variety in Work.

A speaker in a recent dental convention expressed the regret that his practice confined him to operating at the chair almost exclusively, and that "he sometimes felt that he was only a machine wound up each night for an eight hour run the following day." Every practitioner of experience can appreciate the force of this remark, and enter fully into the speaker's conclusion that, "there is nothing so enervating to a dentist as the tiresome and monotonous work at the chair." It reminds us of an incident we recently noticed in a newspaper, that a certain comedian has just completed 2,000 nights of singing comic songs in the same theatre. He was asked if he did not get tired of doing the same thing over and over, smiling before he sang, while he sang, and after he had completed his stunt. His reply was, "No, because my audiences are never the same and they never take my performances alike; but more than this, I like my work and I try to excel." Is this not really the reason the operative dentist, if he be in earnest about his work, does not tire of his calling? There is enough of variety in people and cases to make one who is desirous of excelling in his art to think deeply in the handling of the majority of the cases treated. Patients afford an almost endless variety of moods, and they sometimes tax our ingenuity and diplomacy to keep on friendly terms with them. And who that has won a victory of this sort cares for the backache that follows the strenuous effort involved, and who ever thinks of the brain fatigue or muscle tire of the long and tedious operations when the result is up to one's ideals, and when the appreciative remarks of his patient brush away the brain and muscle fag? The practice of dentistry need not be routine, in fact there is no reason why it should be, unless one is content to do only one small part of practice and to do that by one method. We have known dentists who never mix with other dentists, neither to they read the literature of their profession. How could such men do otherwise than fall into a routine method of practice, and what else could be expected than that they think slightingly of their calling? If we would get the greatest joy and pleasure as well as the most comfort out of our occupation we must put our best thought and endeavor i to it. We can not conclude that we know all that is necessary, or that we are as skilled as the best and need no communion with our fellows, nor that our patients will ever require more of us than they have in the past. The world is moving on and upward every day, and the dentist who does not move with it, will be cast off and lose his opportunity for a place in the "cheering section," at the end of the game. We have got to keep our places in the great game, and happy will be the man who realizes and acts on this fact. Such men will get tired of course, but they will soon get rested and will take up the work again with renewed energy and with ever-increasing skill and inspiration. There are lots of opportunities to vary one's practice, if one line doesn't seem to fit your temperament, try another, until you strike your gait, and then you will run your race with profit as well as pleasure.

**Lost Time.**

In a discussion of a paper on this subject in one of our contemporaries it seems to have been the concensus if not the unanimous opinion of all present, that we waste about as much of our valuable time as we utilize. Instances were cited, too numerous to mention here, as to how we do not economize our time in the conduct of practice, and each speaker seemed to think it possible to devise a code of rules that would fit all men's methods and meet the usual emergencies. One suggested order or system in receiving and handling patients and accidental callers or intruders; another trained assistants, two or three of them for each operator; another modern and complete up-to-date equipment of office in furniture and instruments, etc. We have no doubt all these things will help to save time for some men, and for others they will be equally confusing and wasteful. We have known some persons to be so careful to put into action a system that they lost sight of the main object, or gave so much thought and energy to the running of the system that they made a failure of the treatment. We are all to some extent different, and what is meat for one, may be poison for another. A system that one man could work successfully and greatly to the advantage of his patients, might prove utterly disastrous in the hands of another, whose habit of mind is individual and whose methods of practice have grown up under this peculiarity. We do not want to discourage discussion of this subject, as we certainly believe it has a place in modern business life. But we do feel that if a man is to do his best work, his individuality must predominate over any machine methods. The tools are good, in fact invaluable, but they must fit *us*, or *we* can't use them to advantage.

### Another Year.

With this issue of the DENTAL REGISTER we close the sixty-second volume and year of recorded dental history. We are not claiming to have recorded all the happenings of the past year but we have done what we could to place on record that which has come under our observation that seemed valuable and worth recording, and which might not, except for us, be preserved. We have reprinted much from our exchanges, which we have believed worthy of the widest diffusion, and which might not otherwise come to the notice of our readers. We are conscious of the fact that many excellent ideas have escaped our notice, but believe we have kept our readers in touch with the more important advances of the profession during the year. A glance over the volume index in this issue will convince our most careful readers that our profession has broadened considerably in recent years, and that to keep pace with its advances one must read more than one dental journal. The year 1908 has produced nothing startling. The cast gold inlay has been the most prolific topic of discussion, oral prophylaxis, oral hygiene in public institutions, and the treatment of pyorrhea alveolaris have been much discussed. Two new works on Operative Dentistry have appeared, both from eminent practitioners and teachers, and other text books of value in a literary and professional way have added to our store of recorded knowledge. In a general professional way the re-organization of our National and State Societies gives promise of greater activity in this direction the coming year. Considerable advance in educational methods and standards have been made during the year and more is promised. We hope to fill our place in the New Years work.

